# Familial early puberty: presentation and inheritance pattern in 139 families

**DOI:** 10.1186/s12902-016-0130-x

**Published:** 2016-09-13

**Authors:** Adélaïde Durand, Anu Bashamboo, Ken McElreavey, Raja Brauner

**Affiliations:** 1 Fondation Ophtalmologique Adolphe de Rothschild and Université Paris Descartes, Paris, France; 2 Human Developmental Genetics, Institut Pasteur, Paris, France

**Keywords:** Advanced puberty, Autosomal inheritance, Bilineal inheritance, Central precocious puberty, Early puberty, Familial puberty, GnRH, Hypothalamic-pituitary-gonadal axis, Precocious puberty, Unilineal inheritance

## Abstract

**Background:**

The mechanism that initiates the onset of puberty is largely unknown but the age of onset is mainly under genetic control and influenced by environmental factors including nutrition. The coexistence in the same family of central precocious puberty and advanced puberty, both representing early puberty, suggests that they may represent a clinical spectrum of the same trait due to early activation of the GnRH pulse generator. We therefore evaluated the mode of inheritance of early puberty in a large series of familial cases.

**Methods:**

A retrospective, single center study was carried out on 154 probands (116 girls and 38 boys), from 139 families seen for idiopathic central precocious puberty (onset before 8 years in girls and 9–10 years in boys, *n* = 93) and/or advanced puberty (onset between 8 and 10 years in girls and 10 and 11 years in boys, *n* = 61) seen over a period of 8 years.

**Results:**

Of the 139 families, 111 (80.4 %) had at least one affected 1st degree relatives, 17 (12 %) had only 2nd, 5 (3.6 %) only 3rd and 3 (2.2 %) had both 2nd and 3rd degree affected individuals. In the two remaining families, the unaffected mother had affected girls from two unaffected fathers. In the majority of families the inheritance of the phenotype was consistent with autosomal dominant mode of transmission with incomplete penetrance. An exclusively maternal mode of transmission could be observed or inferred in 83 families, paternal in only 2 families (*p* < 0.0001) and both maternal and paternal modes in 15 families.

In the 139 families, 374 cases of early puberty were identified of whom 315 (84.2 %) were affected females and 59 (15.8 %) affected males (*p* < 0.0001). Twenty one percent of families had exclusively precocious puberty, 25 % had exclusively advanced puberty and 54 % had combinations of both.

**Conclusions:**

The data confirm the high incidence of affected girls with familial early puberty. The mode of inheritance of the phenotype is predominantly maternal. More than half of the families included both precocious and advanced puberty suggesting similar genetic factors.

## Background

Central precocious puberty (PP) is defined as the development of sexual characteristics before the age of 8 years in girls and 9–10 years in boys and this is due to the premature activation of the hypothalamo-pituitary-gonadal axis [1]. Girls are affected more frequently than boys, and in girls the PP is usually idiopathic. Advanced puberty (AP) is defined as the onset of puberty in girls at 8–10 years and in boys at 10–11 years.

The mechanism that initiates the onset of puberty is largely unknown but the age of onset is mainly under genetic control and influenced by environmental factors including nutrition [2, 3]. Although obesity can contribute to early puberty, genetic factors are the strongest predictors of early puberty in different world populations [4–6]. Evidence to support a genetic cause of PP is suggested by familial clustering with up to 27.5 % of central PP cases are familial [7]. The percentage of familial cases may actually be higher because some patients defined as sporadic may have had family members with AP/PP in previous generations and furthermore patients with AP are often excluded from studies. PP and AP may represent a clinical spectrum of the same trait due to early activation of the GnRH pulse generator [7].

Understanding the causes of PP is important since it is reported to be linked with increased risk of other diseases including polycystic ovaries, metabolic diseases and cancer [8, 9]. In addition, the cause of early puberty may possibly determine the evolution of the condition and also help with the therapeutic indications [10]. Known genetic causes of PP are rare. Although mutations involving kisspeptin and its receptor and more recently *MKRN3* are associated with PP, other rare genetic variants in genes implicated in the regulatory mechanism of GnRH secretion such as *TAC3*, *TACR3*, *LIN28B*, *GABRA1* and *NPY* are difficult to link with PP since unaffected family members can carry the variant or functional studies fail to show altered biological activity of the mutant proteins [11–13].

Here, we describe a series of 154 probands from 139 families seen consecutively by the same senior pediatric endocrinologist for PP or AP. Our data confirm previous observations of an increased frequency of affected girls compared with boys and furthermore they show a striking maternal mode of transmission of the phenotype.

## Methods

### Patients

This retrospective single-center cohort study was carried out on 154 probands (116 girls and 38 boys) from 139 families, seen for early puberty (93 PP and 61 AP) with a familial component by a senior pediatric endocrinologist (R Brauner) in a university pediatric hospital from April 2004 to April 2012 (over 8 years). All familial forms evaluated in this period were included. In girls, central PP was diagnosed by breast development before the age of 8 years accompanied by the presence of pubic or axillary hair, a growth rate greater than 2 standard deviation score (SDS) the year before clinical evaluation and/or a bone age (BA) more than 2 years above the chronological age [14]. In boys, PP was diagnosed by pubic hair development associated with testicular enlargement (testicular volume index i.e. length × width > 4 cm^2^) before 9–10 years [15]. We choose this definition as testicular enlargement is not as obvious as the other signs of puberty. AP was diagnosed by the appearance of these clinical signs in girls between the ages of 8 and 10 years and in boys between the ages of 10 and 11 years.

### Transmission

Familial history of early puberty was defined as the age mothers undergoing menarche (data available for all probands with the exception of 3 cases of PP and 2 of AP) before the age of 11.5 years and/or the presence of an early onset of puberty in a family member [7]. The pedigree, timing of puberty in family members and family medical history were established at the first medical visit.

The transmission of the phenotype was analysed according to the pedigrees, which could include one or more probands. First-degree relatives were defined as mother, father, brother(s) and sister(s); second-degree relatives as grandparents, aunt(s) and uncle(s); and third-degree relatives as cousins. The families with at least one first-degree relative affected were further subdivided into three groups: 1) unilineal families (only one affected parent and no evidence for early puberty in the other parent or his/her first-degree relatives); 2) bilineal families (either both parents affected or an unaffected parent having an affected sibling or parent); and 3) families with unaffected parents (one or more siblings affected). Families with lineal maternal inheritance are defined as those in which at least one member of the mother’s family is affected with no evidence of early puberty in the paternal side of the family, with similar definition for lineal paternal inheritance. Uninformative families are those where the phenotype could have been inherited from either side of the family or where the sex of the relatives that transmit the phenotype was unknown.

Autosomal dominant transmission is characterized by the presence of the phenotypes in two successive generations or more. It is characterized with incomplete penetrance if a generation has been skipped. Autosomal recessive transmission included only sibs being affected and both parents being unaffected, although this can also be interpreted as due to *de novo* and dominant mutations.

### Methods

The initial clinical evaluation included determining - the age at the onset of puberty, the height chart, the growth rate, the weight, the pubertal stage and the BA. In all but 8 PP cases and in 43/61 AP, the clinical examination also included an evaluation of the hypothalamic-pituitary-gonadal axis by measuring basal and gonadotropin hormone releasing hormone (GnRH) (100 μg/m2; maximum dose 150 μg)-stimulated luteinising hormone (LH) and follicule-stimulating hormone (FSH) peaks and plasma concentrations of estradiol in girls and testosterone in boys. Pelvic ultrasound examination was performed in 84 (72.5 %) of the girls to evaluate the estrogenic stimulation of the uterus, as an aid in determining the need for GnRH analog treatment. Magnetic resonance imaging (MRI) was performed to exclude a hypothalamic-pituitary lesion. It was not performed in 30 patients with PP and in 38 patients with AP because they had normal neurological evaluation, were aged more than 6 years at onset of puberty in girls and 9 years in boys, and had low plasma estradiol (<15 pg/mL) [16] or testosterone (<0.5 ng/mL) concentrations. The plasma 17-hydroxyprogesterone and testosterone concentrations were measured in all boys and in the girls with pubic or axillary hair development as the first sign of puberty. This was performed to exclude abnormal androgen secretion and congenital adrenal hyperplasia [17]. Boys also underwent an adrenocorticotrophin hormone test. The plasma thyroxin and thyroid-stimulating hormone concentrations were measured to exclude hypothyroidism, and 24 h urinary cortisol was measured to exclude hypercortisolism in those who were overweight or had a rapid weight increase.

Age at the first evidence of puberty was reported by the parents and subsequent studies are described in detail elsewhere [6]. Height, growth rate and body mass index (BMI, weight in kg/height in m squared) were expressed as SDS for chronological age and the pubertal stage was rated according to Marshall and Tanner [18, 19]. BA was assessed (by R Brauner in all) using the Greulich and Pyle method [20]. Plasma LH, FSH, testosterone and estradiol concentrations were measured with RIA. The following values were considered to be pubertal: uterus length ≥35 mm, LH/FSH peak ratio after GnRH test ≥ 0.66 in girls and ≥ 2 in boys [21], and plasma estradiol concentrations ≥ 15 pg/mL and testosterone > 0.5 ng/mL respectively.

Results were expressed as the means ± SD. Statistical analyses were performed using the Student test. Percentages were compared by the Chi-squared test.

## Results

We identified 154 familial cases from 139 families with central idiopathic early puberty. This included 93 probands (68 girls and 25 boys) with PP and 61 probands (48 girls and 13 boys) with AP (Tables 1 and 2).

**Table 1:** Characteristics of the girls with early puberty at presentation

	PP (*n* = 68)		AP (*n* = 48)		Total (*n* = 116)	
	n	m ± SD	n	m ± SD	n	m ± SD
Age at onset, years	66	6.5 ± 1.8	48	8.6 ± 0.6	114	7.4 ± 1.8
Age at evaluation, years	66	7.4 ± 1.9	47	9.4 ± 1.1	113	8.2 ± 1.9
Height, SDS	65	2.1 ± 1.3	47	1.2 ± 1.6	112	1.7 ± 1.5
BMI, SDS	65	1.1 ± 1.2	47	0.7 ± 1.5	112	0.9 ± 1.4
BA advance, years	61	1.2 ± 1.1	36	0.7 ± 1.4	97	1 ± 1.2
Tanner breast	66	2.7 ± 0.7	44	2.8 ± 0.9	110	2.8 ± 0.9
Tanner pubic hair	65	2.1 ± 1.1	42	2.4 ± 1.1	107	2.3 ± 1.1
Age at M mothers, years	66	11.7 ± 1.5	47	11.4 ± 1.6	113	11.6 ± 1.5
Age at M, years	12	10.9 ± 1.8	12	10.7 ± 2	24	10.8 ± 1.9
Uterus length, mm	50	36.5 ± 9	34	37.2 ± 11.5	84	36.8 ± 10
LH peak, IU/L	63	12.2 ± 18.7	33	12 ± 11.5	96	12.1 ± 16.5
FSH peak, IU/L	63	11 ± 5.1	33	9.8 ± 3.4	96	10.6 ± 4.6
LH/FSH peak ratio	63	1 ± 1.1	33	1.2 ± 1	96	1.07 ± 1.1
Estradiol, pg/mL	64	11.5 ± 18	39	12.3 ± 12.3	96	11.8 ± 16
GnRH analog treatment	68	16	46	6	114	22 (19,3 %)

*PP* Central precocious puberty, *AP* Advanced puberty, *M* menstruation

**Table 2:** Characteristics of the boys with early puberty at presentation

	PP (*n* = 25)		AP (*n* = 13)		Total (*n* = 38)	
	*n*	m ± SD	*n*	m ± SD	*n*	m ± SD
Age at onset, years	25	8.8 ± 0.6	12	10.7 ± 0.5	37	9.5 ± 1.1
Age at evaluation, years	25	9.8 ± 1	13	11.4 ± 1.3	38	10.3 ± 1.3
Height, SDS	25	2.2 ± 1.6	12	0.1 ± 1.3	37	1.6 ± 1.8
BMI, SDS	24	1.2 ± 1	12	0.2 ± 1	36	0.9 ± 1.1
BA advance, years	24	1.4 ± 1.2	10	0.3 ± 1.1	34	1.1 ± 1.3
Tanner pubic hair	24	3 ± 1	11	2.8 ± 1.2	35	3 ± 1
Testicular volume, mm	21	36.4 ± 9.3	10	39.8 ± 7	31	38 ± 8.6
Age at M mothers, years	23	11.3 ± 1.5	13	11.4 ± 1.5	36	11.4 ± 1.5
LH peak, IU/L	22	14.7 ± 10.5	10	15.9 ± 5.2	32	15.1 ± 9.1
FSH peak, IU/L	22	5.4 ± 5.3	10	4.8 ± 5.6	32	5.2 ± 5.3
LH/FSH peak ratio	22	3.4 ± 2.7	10	6.1 ± 4.4	32	4.2 ± 3.5
Testosterone, ng/mL	25	1.8 ± 2.7	12	1.1 ± 1.1	37	1.6 ± 2.3
GnRH analog treatment	25	2	13	2	38	4 (10 %)

*PP* Central precocious puberty, *AP* Advanced puberty, *M* menstruation

### Characteristics of the patients

In girls, the age at onset of puberty was < 3 years in 5 (4.3 %) patients, 3–7 years in 22 (19 %), 7–8 years in 41 (35.3 %) and 8–10 years in 48 (41.4 %). In boys, it was 7.5 years in one (2.7 %) patient, 8–10 years in 24 (63.1 %) and 10–11 years in 13 (34.2 %).

In the majority of girls, the first sign of puberty was breast development, although in 3 girls the first sign was pubic hair development followed by breast development before 8 years. In the boys, the mean testicular volume was pubertal at the initial evaluation except in 3 PP boys and one boy with AP who presented with pubic hair development followed by testicular enlargement before 9–10 years in PP or at 10–11 years in AP. The LH/FSH peak ratio was pubertal in 48 girls (50 % of those evaluated) and 22 boys (68.7 %).

### Mode of inheritance

**Fig. 1:**
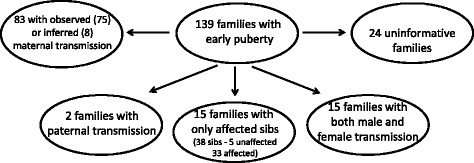
Familial clustering patterns of 139 families with familial early puberty

**Fig. 2:**
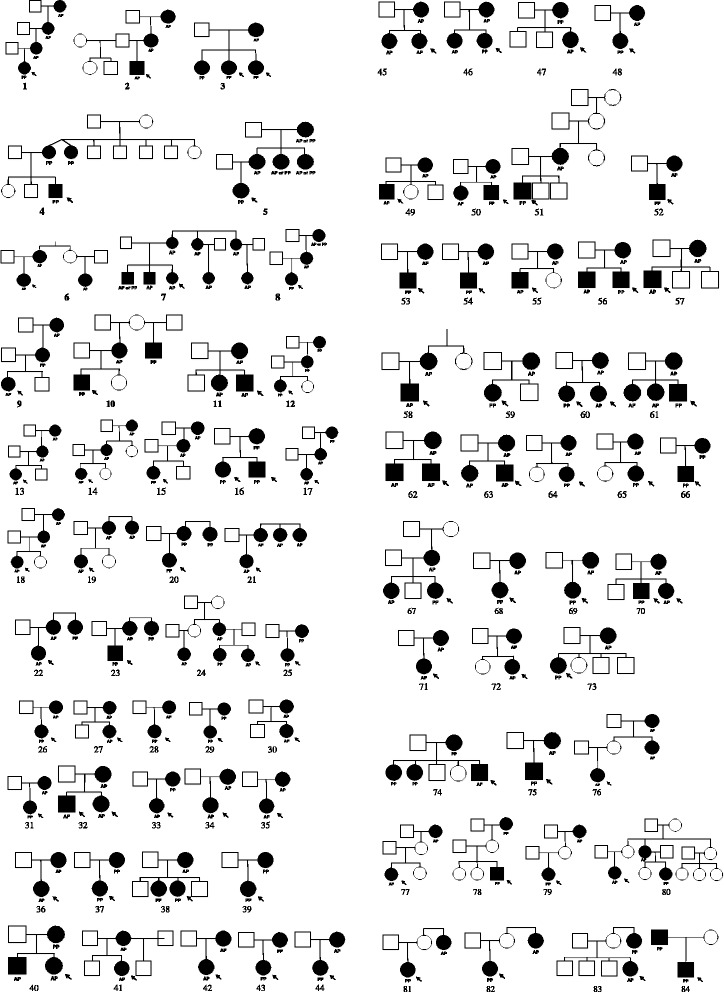
One hundred thirty nine families with central precocious puberty (PP) and/or advanced puberty (AP). Solid squares represent affected male individuals and solid circles affected female individuals. The probands are indicated by an arrow in each case. Bifurcated vertical line indicates non-identical dizygotic twins. Diamond indicates that the sex was unspecified. Pedigrees 1 to 83 are consistent with exclusively maternal inheritance. Pedigree 84 exclusively paternal inheritance

**Fig. 3:**
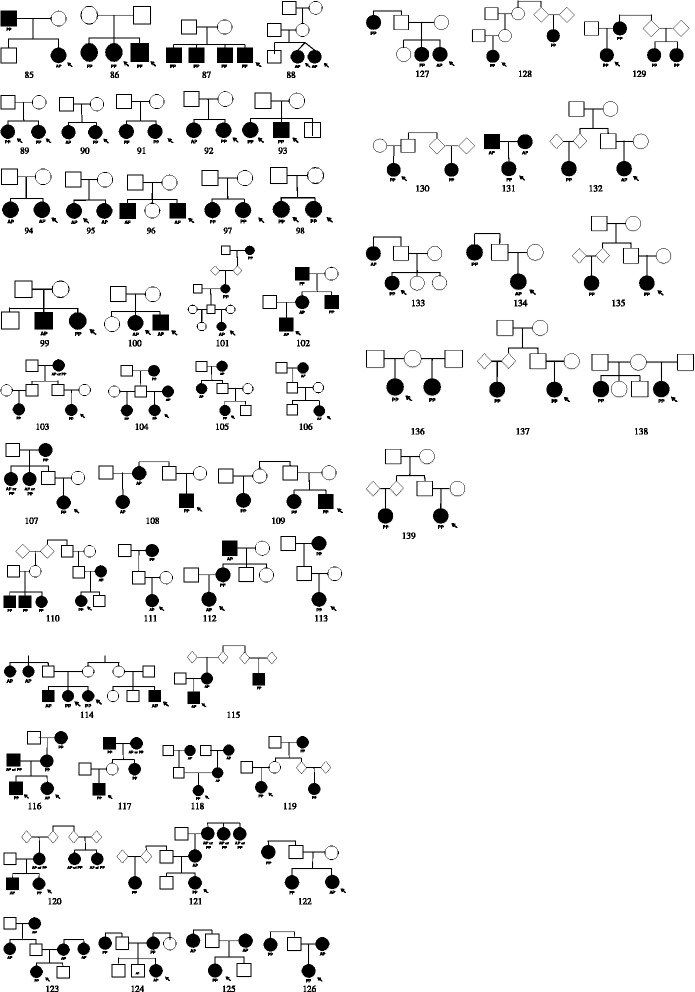
One hundred thirty nine families with central precocious puberty (PP) and/or advanced puberty (AP). Solid squares represent affected male individuals and solid circles affected female individuals. The probands are indicated by an arrow in each case. Bifurcated vertical line indicates non-identical dizygotic twins. Diamond indicates that the sex was unspecified. Pedigree 85 exclusively paternal inheritance, pedigrees 86 to 100 include only affected sibs, and pedigrees 101 to 115 show transmission through both male and female inheritance lineages. Pedigrees 116 to 139 are uninformative in terms of transmission

None of the families reported a history of consanguinity. Familial clustering is represented in Fig. 1 and pedigrees of the 139 families in Figs. 2 and 3.

When the families were classified according to the degree of the transmission of their phenotype, 111 (80.4 %) had at least one affected 1st degree relative (with grandparent affected in 15 families), 17 (12 %) had only 2nd, 5 (3.6 %) only 3rd and 3 (2.2 %) had both 2nd and 3rd degree relatives affected. In one family a 3rd degree cousin was affected. In the 2 remaining families (families 136 and 138), the unaffected mother in each family had affected girls from two unaffected fathers.

In the 111 families with at least one affected 1st degree relatives, at least one parent was affected in 93 families (families 1–75, 84, 85, 102, 104, 110, 112, 115, 116, 118, 120, 121, 123–127, 129 and 131). The high prevalence of the phenotype in parents is strongly suggestive of an autosomal dominant mode of transmission. In 15 families, only sibs were affected; there was no reported family history of PP or AP in the parents or in previous generations (families 86–100). In these families there was a total of 38 sibs of whom 5 were unaffected and 33 affected. It is possible that these families have an autosomal recessive form of the condition but it is important to note that consanguinity was not reported in any of these families and *de novo* mutations may contribute to the phenotype.

The crude penetrance rate was calculated by including the data from all obligate or potential carriers. This identified 72 individuals of which 26 were reported as having the phenotype of either PP or AP. This gives a penetrance value of 36 %.

Among the 93 families with at least one affected parent, we determined if the inheritance of the phenotype was through maternal or paternal lines. Pedigrees consistent with exclusively maternal inheritance was observed in 75 families (families 1–75) and exclusively paternal inheritance was observed in 2 families (84, 85; *p* < 0.0001). The remaining 16 families were uninformative for the mode of transmission of the phenotype.

When one includes all affected 1st, 2nd and/or 3rd degree relatives, the mode of transmission of the phenotype is consistent with exclusively maternal in 83 families (families 1–83), paternal in 2 (families 84 and 85) and through both male and female lineages in 15 families (families 101–115). The age at the onset of puberty and clinical characteristics are not different from those families showing unilineal and bilineal inheritances. Other more complex modes of transmission of the phenotype are possible for these pedigrees. For example, imprinting anomalies may contribute to the phenotype in families 103–106. Families 116–139 were uninformative concerning the mode of transmission of the phenotype.

Of the 154 probands (116 girls and 38 boys) with familial early puberty, the overall female to male ratio was 3.1:1. Of the 139 families, a total of 374 cases of early puberty were identified of whom 315 (84.2 %) were affected girls and 59 (15.8 %) affected boys (Chi-squared 175.230; *p* < 0.0001). The overall female to male ratio was 5.3:1. When the 125 probands, who had at least one affected 1st degree relative (91 girls and 34 boys), and their familial affected members (145 girls and 18 boys) were analysed together, 236 (81.9 %) were affected girls and 52 (18.1 %) affected boys (*p* < 0.001). The overall female to male ratio was 4.5:1.

A total of 374 cases presented with early puberty, of these 21 % of the families had exclusively PP, 25 % had exclusively AP and 54 % had both PP and AP suggesting that both phenotypes are part of a spectrum. In the subgroup of families showing maternal inheritance, 16.5 % exclusively PP, 31.5 % had exclusively AP and 52 % of families had both AP and PP. Although there is a tendency towards AP with a lower incidence of PP in the families showing maternal inheritance of the phenotype, this is not statistically significant.

## Discussion

This is the first study examining children with precocious and advanced puberty. It establishes that they both are present in 54 % of the families studied here. In a previous study analysing 493 consecutive girls seen over 31 years for central PP (including the 68 girls with familial central PP of the present study seen over 8 years), we found 41.4 % (192/465 after exclusion of the adopted) of familial forms [6], while De Vries et al. reported 27.5 % [7]. We found that the familial forms had significantly greater frequencies of pubertal LH/FSH peak ratios and lower plasma estradiol concentrations at the first evaluation than the sporadic forms, while the ages at onset of puberty, number of signs of puberty associated with the breast development and frequencies of GnRH treatment were similar. The BMI SDS was also similar in the familial and in the sporadic forms, suggesting that the familial obesity is not the causative factor in the familial forms. We also found a lower age at first menstruation in the mothers of girls with familial form of CPP than in those with sporadic forms (*p* = 0.0001), as reported by de Vries et al. [7] who found similar age of the mothers in familial CPP than in our population (11.47 vs 11.5 years). Only 5 (4.3 %) of the probands girls were aged less than 3 years at onset of puberty. All had rapidly progressing CPP that required GnRH analog treatment [6]. Only one boy was aged less than 8 years at onset of puberty suggesting that pubertal onset before this age in boys suggests organic CPP [15, 22].

In the majority of families presented here, the inheritance of the phenotype is compatible with an autosomal dominant mode of transmission with incomplete penetrance (36 %) and variable expressivity. We observed three or more affected individuals in successive generations in 15 families (10.8 %), while de Vries et al. [7] reported that in 47.8 % of the central PP cases three generations were affected. The lower frequency reported here is probably due to the fact that de Vries et al. included only families in whom the age at puberty in three generations was known. Similar to previous studies we found a higher incidence of girls compared to boys when all the affected members are included (5.3:1). The striking observation in this study is the degree to which inheritance of PP or AP is transmitted through the maternal lineage. Of the 93 pedigrees with at least one parent affected, 80.6 % had an exclusively maternal inheritance of the phenotype with only two families having exclusively paternal inheritance. When we include all pedigrees, with the exception of the families with only affected sibs (*n* = 15) the values are 66.9 % of families consistent with exclusively maternal inheritance, 1 % exclusively paternal and 12.1 % inherited through both male and female lineages and 19.4 % uninformative. De Vries et al. also found no consanguinity in their 46 families [7]. Recently, Wohlfahrt-Veje et al. [23] by evaluating a longitudinal cohort found that both maternal and paternal timing of puberty had a strong influence on the timing of pubertal onset in children, and this was not sex-specific.

Alterations in genomic imprinting may play a key role in anomalies of pubertal timing. Recently, in a study of 15 families with central PP, 5 families were identified who carried heterozygous frameshift or missense mutations in the *MKRN3* gene that encodes the makorin RING-finger protein 3 [24]. The precise function of MKRN3 is unclear but may be involved in ubiquitin-ligase activity. The *Mkrn3* gene is expressed in the arcuate nucleus of both male and female mice at postnatal day 10 and declined thereafter [25]. The arcuate nucleus expresses a number of key genes involved in pubertal development such as Kisspeptin and TAC2, whose expression increases as the levels of Mkrn3 decline. Thus, *MKRN3* would act as a negative regulator of puberty, with loss-of-function mutations resulting in central PP. Interestingly, *MKRN3* is subject to imprinting and it is expressed exclusively from the paternally inherited chromosome [24]. In total 26 mutations have been described in the *MKRN3* gene, of which 10 are frameshift mutation, 13 missense mutations and 3 nonsense mutations [26–28]. Where it was possible to study all of the affected persons inherited the mutation from their fathers. Here, we observed a predominant maternal inheritance of PP/AP rather than paternal inheritance. This could either be due to the high incidence of early puberty in girls (and hence higher frequency of maternal transmission) or the phenotype may be influenced by transmission of mutations involving a maternally imprinted gene. This could be resolved by the analysis of large and informative familial cases of PP/AP.

## Conclusions

The data confirm the high incidence of affected girls with early puberty compared with boys in familial cases. The mode of inheritance of the phenotype is predominantly maternal. More than half of the families included both precocious and advanced puberty suggesting similar factors.

## Abbreviations

AP: Advanced puberty
BA: Bone age
BMI: Body mass index
FSH: Follicle stimulating hormone
GnRH: Gonadotropin hormone releasing hormone
LH: Luteinising hormone
MRI: Magnetic resonance imaging
PP: Central precocious puberty
SDS: Standard deviation score
